# Biomechanical efficacy of four different dual screws fixations in treatment of talus neck fracture: a three-dimensional finite element analysis

**DOI:** 10.1186/s13018-020-1560-8

**Published:** 2020-02-11

**Authors:** Zhengrui Fan, Jianxiong Ma, Jian Chen, Baocheng Yang, Ying Wang, Haohao Bai, Lei Sun, Yan Wang, Bin Lu, Ben-chao Dong, Aixian Tian, Xinlong Ma

**Affiliations:** 1 Biomechanics Labs of Orthopaedics Institute, Tianjin Hospital, Tianjin, 300050 People’s Republic of China; 2 Tianjin Hospital, Tianjin University, Tianjin, 300211 People’s Republic of China; 3 Department of Orthopedics, Tianjin Hospital, Hexi District, Tianjin City, People’s Republic of China

**Keywords:** Talus neck fracture, Finite element analysis, Three-dimensional reconstruction, Internal fixation

## Abstract

**Background:**

Current there are different screws fixation methods used for fixation of the talar neck fracture. However, the best method of screws internal fixation is still controversial. Few relevant studies have focused on this issue, especially by finite element analysis. The purpose of this study was to explore the mechanical stability of dual screws internal fixation methods with different approaches and the best biomechanical environment of the fracture section, so as to provide reliable mechanical evidence for the selection of clinical internal fixation.

**Methods:**

The computed tomography (CT) image of the healthy adult male ankle joint was used for three-dimensional reconstruction of the ankle model. Talus neck fracture and screws were constructed by computer-aided design (CAD). Then, 3D model of talar neck fracture which fixed with antero-posterior (AP) parallel dual screws, antero-posterior (AP) cross dual screws, postero-anterior (PA) parallel dual screws, and postero-anterior (PA) cross dual screws were simulated. Finally, under the condition of 2400N vertical load, finite element analysis (FEA) were carried out to compare the outcome of the four different internal fixation methods. The results of Von Mises stress, displacement of four groups which contain talus fracture fragments and screws internal fixations were analyzed.

**Results:**

Compared with the other three groups, postero-anterior (PA) parallel dual screws had better results in the stress peak, stress distribution, and displacement of talus and internal fixation.

**Conclusions:**

To sum up, the Von Mises stress of fracture section was the smallest, the stress distribution of screws were the most scattered, and the peak value was the smallest in posterior to anterior parallel double screws fixation, which was obviously better than that in the other three groups. When using screws internal fixation, the method of posterior to anterior screws fixation is better than that of anterior to posterior screws fixation, and the peak value and stress distribution of parallel double screws fixation is better than that of cross double screws fixation. Thus, for the talar neck fracture, the use of posterior to anterior parallel double screws fixation is recommended in clinical surgery.

## Introduction

Talus is fundamental to connect tibiofibula and foot. It has a unique anatomical structure and function. Fractures of the talus are uncommon injuries which caused by high energy injury [1], but talus neck fracture is more common, accounting for 45% of all talus fracture [2]. Although there are many internal fixation methods for talus neck fracture, including Kirschner needle fixation, plate fixation, screw fixation, etc [3], the complications and outcomes after treatment remain challenging for subsequent management. At present, most surgeons usually place screws from anterior through the dual anteromedial and anterolateral approaches [4]. Another method of fixation involves placing the screw from a posterior to anterior position, which is less used because of the difficulty of placement. Antero-posterior screw fixation can fully expose the surgical area. While the postero-anterior screw fixation can reduce the chance of vascular rupture. The stress distribution of different internal fixation is different, the issue of its stability has been a controversial and much-disputed subject within the field of orthopedic surgery [5]. Previous research has established that the growth of bone is closely related to the stress environment of the bone. When stimulated by reasonable stress, the bone cells are more active and the bone-healing ability is stronger [6]. Meanwhile, recent evidence suggest that the initial stability of the fracture end is the key factor for the formation of callus, and a reasonable fixation method can provide good stability for the fracture end and better growth conditions for the talus [7].

At present, there are few biomechanical studies on the mechanical stability of the antero-posterior screw fixation compared with the postero-anterior screw fixation. Debate continues about the best strategies for the internal fixation of talus neck fracture in clinical. Previous studies were limited to applying pressure to a single talus, considering only that the talus bone was subjected to vertical upward shear forces. The test did not construct the surrounding structure of the ankle joint, and the applied load was inconsistent with the physiological load [8]. At the same time, the internal fixation methods used in the experiment are limited, and there has been no detailed investigation of the biomechanical comparison between parallel and cross dual screws.

In recent years, with the wide application of finite element analysis method in the field of orthopedics, it is of great clinical significance to accurately calculate the stress, strain, and displacement of bone under the complex conditions of load, constraint condition, and intraosseous structure. In this paper, three-dimensional finite element analysis was used to analyze the fixation of the antero-posterior parallel and cross dual screws and the postero-anterior parallel and cross dual screws of talus neck fracture. The biomechanical strength of four internal fixation methods and their effects on the mechanical stability of talus fracture were compared.

## Materials and methods

### Three-dimensional modeling and pre-processing

In order to reconstruct the normal ankle joint three-dimensional modeling, the volunteer was asked to undergo CT scans. A healthy young male volunteer was recruited who was 22 years old and had a height of 176 cm and a weight of 60 kg, and had no history of foot trauma, tumor, and anatomical abnormal after clinical examination. The volunteer’ s feet remained neutral position during the CT scan of the ankle joint. The scanned CT data was saved in DICOM format. This study had been approved by the ethics committee of the hospital.

**Fig. 1:**
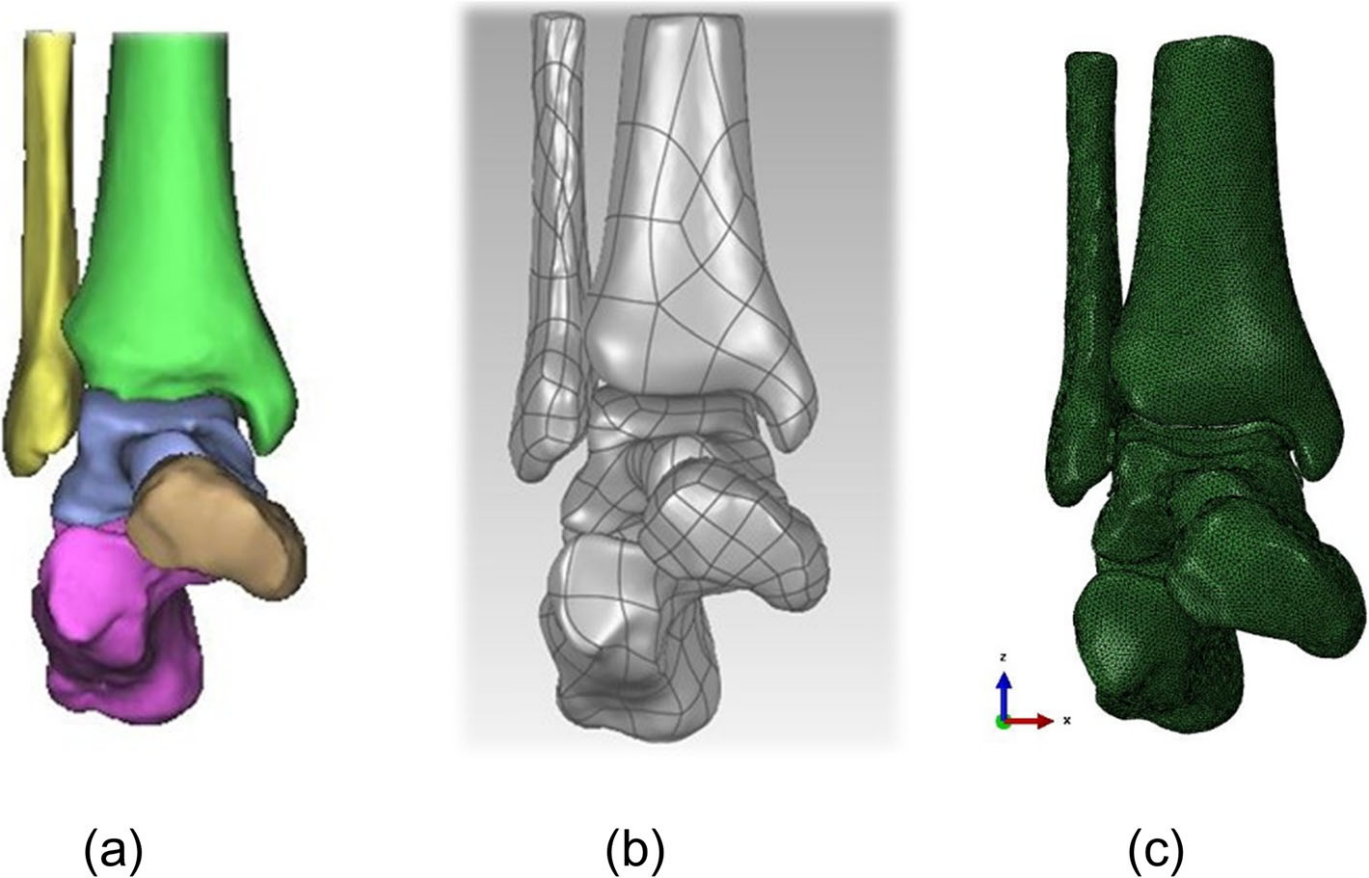
**a** Three-dimensional model reconstruction of ankle joint. **b** Surface fitting of a three-dimensional model of ankle joint. **c** Meshing of the ankle joint model

**Fig. 2:**
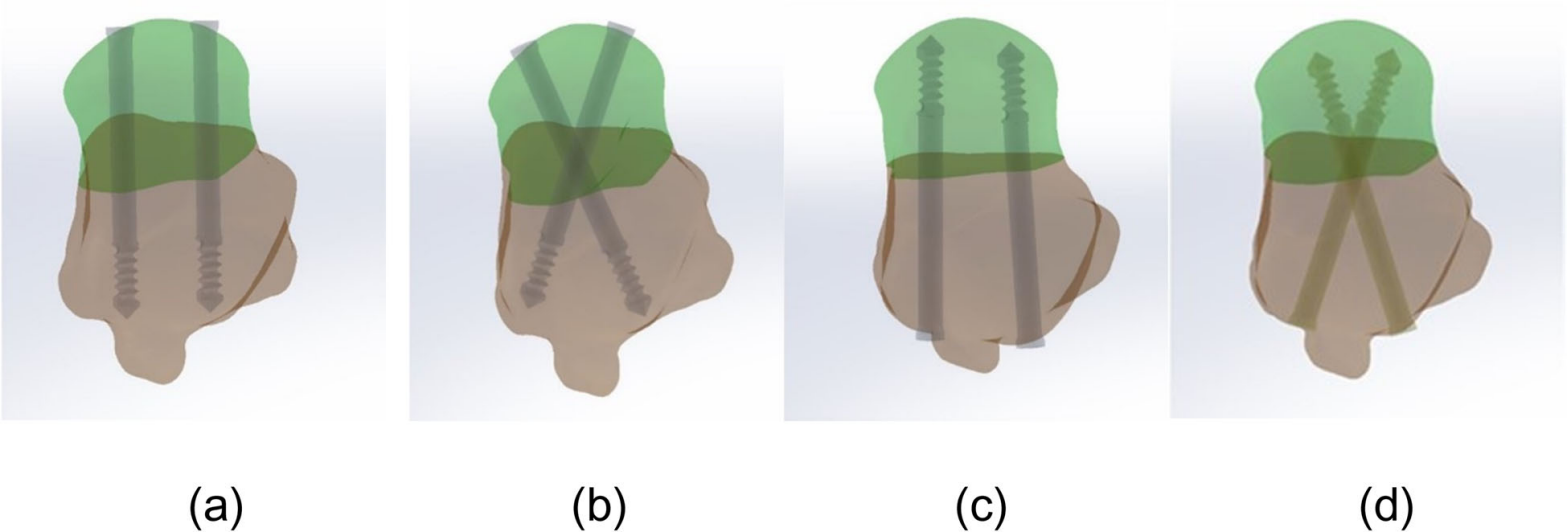
Model of talus neck fracture showing four different fixation strategies. **a** Antero-posterior parallel dual screws internal fixation methods. **b** Antero-posterior cross dual screws internal fixation methods. **c** Postero-anterior parallel dual screws internal fixation methods. **d** Postero-anterior cross dual screws internal fixation methods

The ankle data was imported into Mimics17.0 software in DICOM format (Materialise, Belgium). The three-dimensional surface geometry of the bones reconstruction of peri-ankle bones, including fibula, tibia, talus, calcaneus, and scaphoid, was performed by threshold segmentation, region growth, and manual erasure, etc. (Fig. 1a). Then, each bone of ankle in txt. format was transferred to the reverse engineering software Geomagic Studio 2014 software (Geomagic Company, USA). Processing by unifying, removing of external solitary points, reducing noise, and package and surface fitting were done to acquire the volumes of the bones (Fig. 1b). Meanwhile, the cartilage in corresponding bone space was constructed. All entities were exported in IGES format. Then, the Solidworks software (Dassault, France) was used to simulate the talus neck fracture and generate cancellous-bone lag screws (the outer diameter of the thread is 4.5 mm and the inner diameter is 3.0 mm). Next, the talus and four internal fixation models were sequentially assembled, and the fracture models with the antero-posterior (AP) parallel and cross dual screws and the postero-anterior (PA) parallel and cross dual screws internal fixation methods were generated (Fig. 2). Each assemble was meshed by tetrahedral mesh in the Hypermesh 13.0 software (Altair Engineering, Inc., USA) (Fig. 1c). Finally, three-dimensional models of the ankle with four associated internal fixations in ing format were imported into finite element solution software Abaqus 6.14 (Simulia Corp., USA). And related ligaments around the ankle were constructed through the spring unit.

### Material properties

In Abaqus 6.14, different parts of the models have defined the material properties. In this study, tissue such as the bones, cartilage of the ankle and screws were regarded as homogeneous, continuous, isotropic, and linear elastomers material under quasi-static load. According to relevant literatures [9–11], the stiffness value of ligaments around the ankle joint was 80 N/mm. The material properties [12–14] for each component are shown in Table 1.

**Table 1:** Material properties used in finite element models

Material	Young’s modulus (MPa)	Poisson ratio
Bone	7300	0.3
Cartilage	10	0.4
Internal fixation	110,000	0.3

### Contact setting, constraints, and loads

**Fig. 3:**
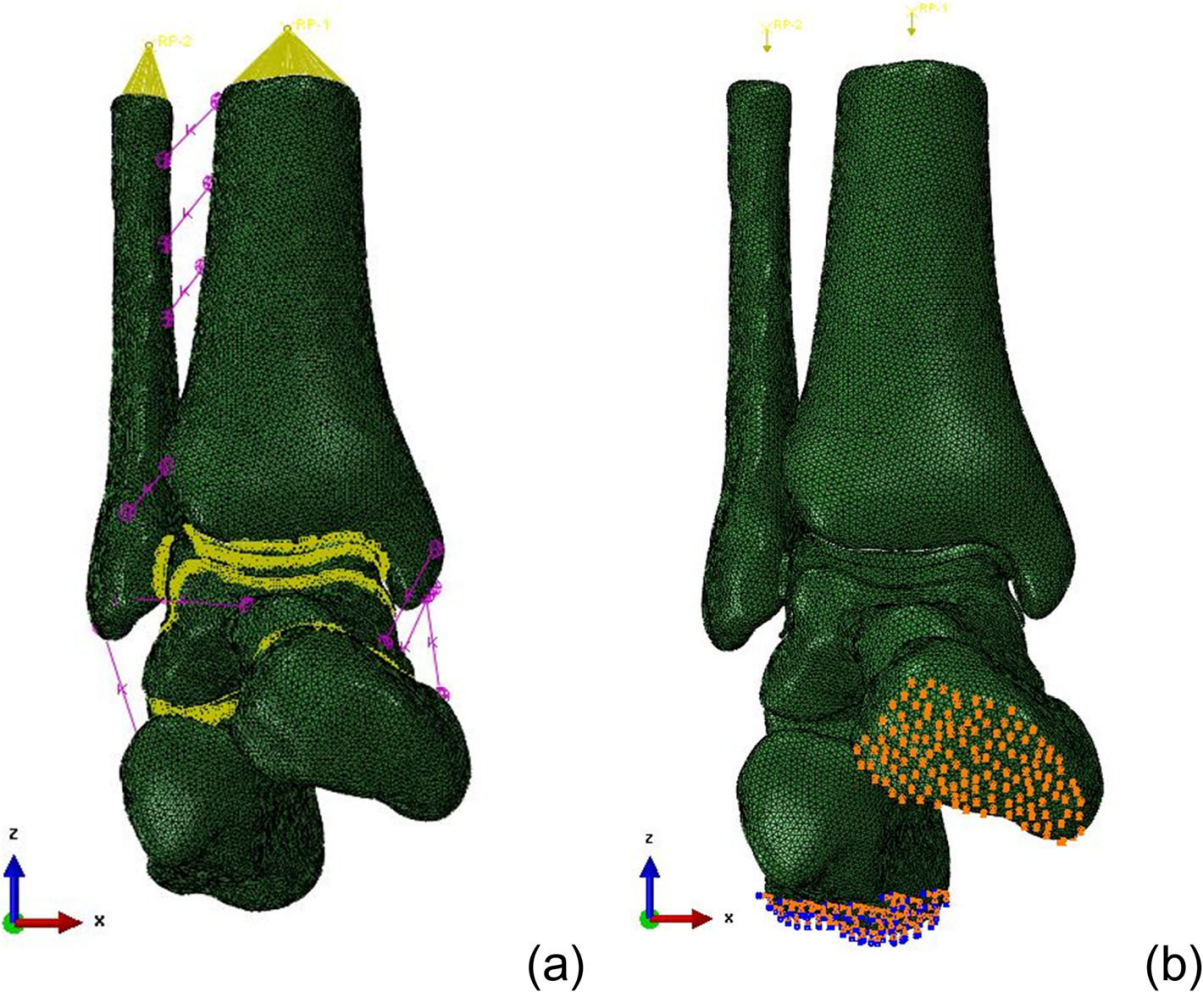
**a** Interaction settings of 3D model. **b** Constraint and load settings of 3D model

According to the contact method described in references [15, 16], in an ankle model containing bones, cartilage, ligaments, and screws, the boundary condition of the contact between the bone surface and the cartilage surface was set to bind, and the start and stop points of ligaments which connect with the bone were set to bind. The transmission of force between bone and bone was realized by contact between cartilages. The contact condition between articular cartilages was set to friction (friction coefficient 0.01) to simulate the relative motion between articular cartilages [17]. In the fracture model, the complete fracture of the talus was simulated, and the fracture surface remained in complete contact after reduction, and the rigid fixation of fracture was realized by simulating screw at the same time. So the fracture surfaces of the talus neck fracture were set to friction (friction coefficient = 0.34). Bind was used between the screws and the bone as shown in Fig. 3a.This study simulated the single-leg standing position, and the constraint condition of the distal end of the calcaneus was set to completely fixed. The scaphoid bone moved freely in the *X*-axis and was completely fixed in the *Y*- and *Z*-axes. A vertical downward load of 2400 N corresponding to 400% body weight was applied along the upper section of the tibia and fibula, and the tibiofibular ratio was 5:1 as shown in Fig. 3. The validity of the model was verified under the condition of 600n load. The results showed that the model was in good consistency with the experimental data in relevant literatures [18], and the model was real and reliable, which could be used for further experimental research.

## Result

The stress distributions, stress peaks, and displacement of the talus and four internal fixations were examined.

### Von Mises stress distribution

**Fig. 4:**
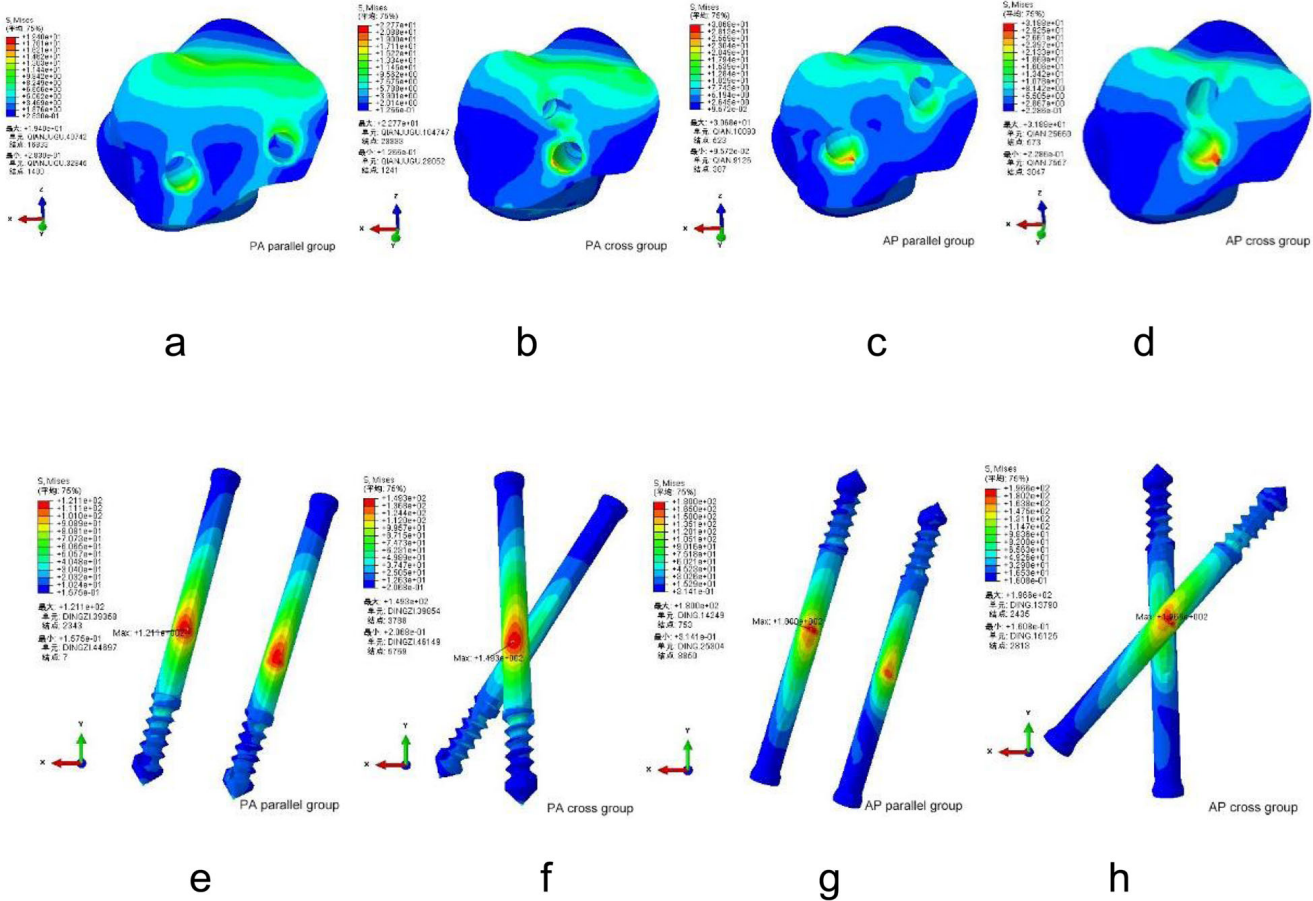
**a**–**d** The stress distribution of talus. **e**–**h** The stress distribution of internal fixation

In four different types of internal fixations, differences in stress distribution were observed. When standing on a single leg, the pressure at the broken end of the fracture was mainly located in the dorsal part of the talus neck and gradually decreased to the metatarsal side. At the end of the talus fracture, the maximum stress was mainly concentrated at the intersection of fracture line and screw of each group. And the peak stresses at the talus were 19.40 MPa, 22.77 MPa, 30.68 MPa, and 31.88 MPa in PA parallel and cross dual screws group, AP parallel, and cross dual screws group (Table 2 and Fig. 4a–d).

**Table 2:** Parameters results

Parameters	PA parallel dual screws	PA cross dual screws	AP parallel dual screws	AP cross dual screws
Maximum talar stress (MPa)	19.40	22.77	30.68	31.88
Internal fixation maximum stress (MPa)	121.10	149.30	180.00	196.60
The maximum displacement of the talar (mm)	1.597	1.674	1.673	1.764
The maximum displacement of the internal fixation (mm)	1.244	1.505	1.495	1.392

In the four internal fixation of screws, the Von Mises stress distribution of the four kinds of screws was uneven, showing the stress at both ends of the screw was low and the stress in the middle was high. The maximum stress was mainly distributed at the middle surface of the screw near the intersection of the fracture line of the metatarsal side of the talus, and the peak Von Mises stresses were 121.10 MPa, 149.30 MPa, 180.00 MPa, and 196.60 MPa in PA parallel and cross dual screws group, AP parallel and cross dual screws group (Table 2 and Fig. 4e–h).

### Model displacement

**Fig. 5:**
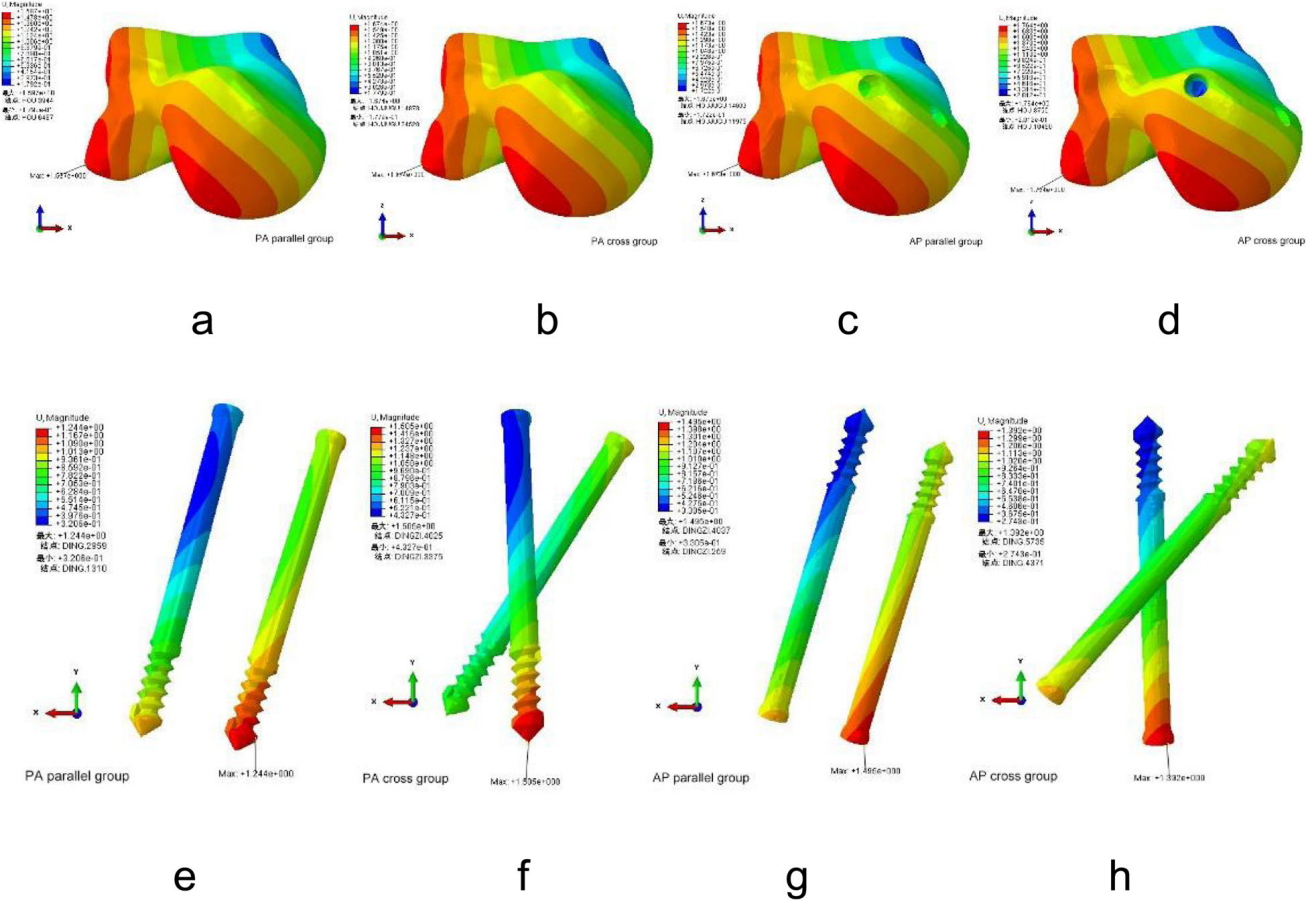
**a**–**d** The displacement of the talus. **e**–**h** The displacement of internal fixation

Analysis of the model displacement showed that PA parallel screws group shows the lowest amount of displacement. The maximum displacement of the talus was located at the lateral processes of the body of the talus and the outer and lower sides of the talus head. The maximum displacements were 1.597 mm, 1.674 mm, 1.673 mm, and 1.764 mm in PA parallel and cross dual screws group, AP parallel, and cross dual screws group (Table 2 and Fig. 5a–d).

According to the displacement contour of the four groups of screws, the maximum dual screws internal fixation displacements were 1.244 mm, 1.505 mm, 1.495 mm, and 1.392 mm in PA parallel and cross dual screws group, AP parallel and cross dual screws group. These data show that the postero-anterior parallel dual screws internal fixation group exhibited lower displacement compared to the other groups (Table 2 and Fig. 5e–h).

## Discussion

Most of the talus neck fractures are caused by deceleration violence with axial impact, accounting for about 45% of all talus fractures. The injury mechanism is due to the sharp extension of the ankle joint and the violent impact of the anterior ankle on the talus neck [3, 19]. Because the talus neck is slender and irregular in structure, it is easy to lead to malunion, nonunion, aseptic osteonecrosis, infection and traumatic ankle or subtalar arthritis after injury [20, 21]. The methods of open reduction and internal fixation for talus neck fracture include posterior to anterior screw fixation, anterior to posterior screw fixation, Kirschner wire fixation, and plate fixation. Strong fixation can maintain the stability of the talus of the fracture, so as to ensure the stability of the surrounding joint. The type, number, fixed orientation of the internal fixation, and the orientation of the fracture line can affect the strength of the internal fixation. The common fixation method is screw fixation, but the anti-rotation ability of single screw is poor, so double screws are the first choice for posterior fixation of talus neck fracture [22]. It is well known that the different fixation directions and fixation methods of screws are not only the main factors affecting the stress distribution of talus, but also the key factors to promote fracture healing. Several reports have shown that proper internal fixation can provide a good mechanical environment for talus neck fracture and improve the cure rate of talus neck fracture [23, 24]. After the fracture of the talus neck was incised and fixed, the stress on the fracture surface is too large, which will lead to an increase in the probability of secondary fracture and even bone resorption. On the contrary, it cannot effectively stimulate bone formation. The maximum Von Mises stress of the four groups in this study was within a reasonable range, in which the maximum stress on the fracture surface of the posterior to anterior parallel screws fixation group was the smallest, and the stress distribution on the fracture section was more uniform than that of the other three groups, which was beneficial to better fracture healing.

Orthopedic surgeons generally treat the talus neck fracture through the anterior or posterior approach [25]. Good reduction and rigid fixation of fracture end can accelerate the process of bone healing, and different internal fixation can stabilize the broken end of fracture. The choice of approach and the way of fixation determine the stress distribution of internal fixation to a certain extent, and the stress state of internal fixation is also the standard to evaluate the fixation effect of internal fixation. Wolff [6] showed that the growth of bone was affected by the surrounding stress environment. When the stress stimulation reaches the best, a dynamic balanced environment can be formed between osteogenesis and osteoclast. When the bone is in a stressful environment that is too large or too small, the balance between osteogenesis and osteoclast is broken, followed by dynamic changes to achieve a new balance [26]. Because too high-stress concentration will usually lead to high strain at the broken end of talus fracture, too low strain will hinder the construction of local callus, so the ideal stress state of internal fixation should meet the conditions of uniform stress distribution and small peak value of stress concentration. For fracture healing, the direction and arrangement of screws are important factors affecting the stress distribution on the contact surface of fracture. In clinical treatment, the selection of treatment is still controversial. The anterior to posterior screw fixation can fully expose the surgical field of vision and is easy to enter the needle, but the screw is located in the eccentric position, which is not conducive to the vertical fracture line and is easy to damage the scaphoid joint. The posterior to anterior screw fixation is minimally invasive, and the screw is located in the center of the talus neck, which can be vertically fractured, but the surgical visual field is not easy to be fully exposed, the nail is difficult to enter, and the blood flow around the talus neck is very easy [27]. In this study, the internal fixation stress distribution of the four groups was more concentrated at the broken end of the fracture. Through the comparison of the stress distribution of the four groups, it was found that the stress concentration of the screw was the smallest when the posterior to anterior parallel double screws were fixed, and the peak value was 121.10 MPA. At the same time, the stress distribution was more dispersed and uniform than the other three groups. The results of this study showed that among the four groups of displacements, the talus and screw displacements in posterior to anterior parallel double screws group were the smallest which indicated that the biomechanical fixation strength of PA parallel double screws was superior to the other three groups.

In recent years, much attention has been paid to the biomechanical research of the foot and ankle. With the continuous development of digital medicine, computer digital simulation technology is more and more used in the simulation of an ankle injury. At present, it has become one of the commonly used methods in orthopedic biomechanics research, which has the advantages of easy to control experimental conditions, low cost, high precision, and so on. Through the digital simulation of four internal fixation methods of talus neck fracture based on the finite element model of ankle joint after three-dimensional reconstruction of CT, the author strives for the optimal fixation mode of talus neck fracture. There are still some limitations to this study. Firstly, during the modeling of the fracture model, the fracture was simplified, and the real fracture line was irregular, but the ankle model met the experimental requirements and had been verified. Secondly, the study only carried on the finite element analysis of the ankle joint after internal fixation of talus neck fracture when standing on one foot, but did not analyze the stress of other loading methods. In the real case, the ankle joint is often affected by a variety of mechanical factors. Third, the ankle model only considered the stress of five bones, corresponding cartilage and ligaments, and did not consider the effect of surrounding muscle, skin, and other soft tissue on the stress of the ankle joint after screw fixation.

## Conclusion

To sum up, the Von Mises stress of fracture section was the smallest, the stress distribution of screws was the most scattered, and the peak value was the smallest in posterior to anterior parallel double screws fixation, which was obviously better than that in the other three groups. When using screws internal fixation, the method of posterior to anterior screws fixation is better than that of anterior to posterior screws fixation, and the peak value and stress distribution of parallel double screws fixation is better than that of cross double screws fixation. Thus, for the talar neck fracture, the use of posterior to anterior parallel double screws fixation is recommended in clinical surgery.

## Data Availability

The original CT data used in this study belongs to the asset of the hospital. The authors may request the hospital to share the data on reasonable request.

## Abbreviations

AP: Antero-posterior
CAD: Computer-aided design
FEA: Finite element analysis
PA: Postero-anterior
