# Correction to: Biomechanical efficacy of four different dual screws fixations in treatment of talus neck fracture: a three-dimensional finite element analysis

**DOI:** 10.1186/s13018-022-03036-1

**Published:** 2022-03-11

**Authors:** Zhengrui Fan, Jianxiong Ma, Jian Chen, Baocheng Yang, Ying Wang, Haohao Bai, Lei Sun, Yan Wang, Bin Lu, Ben-chao Dong, Aixian Tian, Xinlong Ma

**Affiliations:** 1 Biomechanics Labs of Orthopaedics Institute, Clinical College of Orthopedics, Tianjin Medical University, Tianjin, 300050 People’s Republic of China; 2 Tianjin Hospital, Tianjin University, Tianjin, 300211 People’s Republic of China; 3 Department of Orthopedics, Tianjin Hospital, Hexi District, Tianjin City, People’s Republic of China

## Correction to: Journal of Orthopaedic Surgery and Research (2020) 15:45 10.1186/s13018-020-1560-8

Following publication of the original article [1], the authors identified an error in the first affiliation of the authors.

The correct affiliation should be changed from “^1^Biomechanics Labs of Orthopaedics Institute, Tianjin Hospital, Tianjin, 300050 People’s Republic of China” to “^1^Biomechanics Labs of Orthopaedics Institute, Clinical College of Orthopedics, Tianjin Medical University, Tianjin, 300050 People’s Republic of China”.
